# The clinical efficacy and patient satisfaction of the virtual fracture clinic in the UK: a systematic review

**DOI:** 10.1186/s13018-025-06418-3

**Published:** 2025-11-10

**Authors:** Joseph William Johnson, Abdul-Hadi Kafagi, Anand Pillai

**Affiliations:** 1 Faculty of Biology, Medicine and Health, The University of Manchester, Manchester, UK; 2 Department of Trauma and Orthopaedics, Wythenshawe Hospital, Manchester University Hospitals NHS Foundation Trust, Manchester, UK

**Keywords:** Virtual fracture clinic, Fracture, Clinical efficacy, Patient satisfaction, Patient reported outcomes, Telemedicine, Telehealth, Trauma, Virtual care, Remote consultation

## Abstract

**Background:**

Virtual fracture clinics (VFCs) have been proposed as an efficacious alternative to face-to-face (FTF) fracture clinics. Better usage of clinical time and resources, increased accessibility, decreased patient wait times and reduced cost may be advantages of the VFC. This study aims to assess the clinical efficacy and patient satisfaction of the VFC in the UK since introduced in 2011 and determine whether VFCs should become the leading pathway for acute traumatic orthopaedic (ATO) care.

**Methods:**

A systematic review of studies from 2011 up to April 2025 was conducted, identifying all relevant literature to the safety and efficacy of VFCs. MEDLINE, PubMed and the Cochrane library were searched according to our search strategy. Systematic screening and application of our inclusion and exclusion criteria resulted in twenty-five included studies. These studies included: retrospective cohort studies, case series and interrupted time series, prospective and cross-sectional cohort studies, closed loop audits and service evaluations. Key clinical efficacy outcomes assessed included: VFC discharge rates, adherence to BOAST 72-h guidelines, missed injuries, inappropriate referrals/radiographs, reattendances, and the number of FTF fracture clinic follow ups following VFC review. Additional outcomes assessed were patient reported outcome measures and patient satisfaction data.

**Results:**

A cohort of 63,367 patients contributed to the clinical efficacy summative outcomes. VFCs reported an 83.6% mean compliance rate with British Orthopaedic Association Standards for Trauma 72-h guidelines, compared to 5.7% for FTF fracture clinics. VFCs make minimal diagnostic errors and as a result reported a low mean reattendance rate following discharge of 4.9%. Patients have good health outcomes, a high mean satisfaction rate of 85.4% for VFCs and prefer them to FTF fracture clinics.

**Conclusion:**

This systematic review demonstrates that VFCs are highly efficacious in managing ATO patients in the UK and that patients are satisfied with their care. VFCs outperform FTF fracture clinics and could become the standard pathway for management of ATO injuries across the UK in the future. In the meantime, further research is needed to validate these comparisons between VFCs and FTF fracture clinics to definitively conclude that VFCs should become the leading pathway.

**Supplementary Information:**

The online version contains supplementary material available at 10.1186/s13018-025-06418-3.

## Introduction

The fracture clinic is an outpatients department that is used to assess and manage patients who have been recently diagnosed with bone fractures. The traditional face-to-face (FTF) fracture clinic has posed several issues such as an inability to meet current patient demand due to a limited number of clinical appointments, significant cost through time and resources as well as often having to rely on junior members of staff to make clinical decisions to try to meet guidelines [1].

The virtual fracture clinic (VFC) is an adaptation of the FTF fracture clinic and now favoured within the UK, where follow up care and management is provided remotely [2]. Typically, patients diagnosed with bone fractures are referred to the VFC from the emergency department or the minor injuries unit (MIU) [2]. From here, most cases are discharged home with knowledge of the nature of their injury, immediate management such as splinting and analgesia as well as an appointment within 72 h at the VFC to discuss their next steps [2]. The VFC consists of an orthopaedic consultant reviewing radiographs and patient notes to formulate a management plan for the patient, which is communicated via a telephone call from a trauma specialist nurse [2, 3]. The management options available for patients following the VFC include discharge with advice but no further review, a review in a sub-specialty fracture clinic or an urgent review in person for complex issues [2, 3]. The primary goals of VFCs are to improve patient care and experience through reduction in travel, increased accessibility and decreased waiting times, as well as to reduce the strain of the increasing numbers of patients referred to the fracture clinic and cost savings [4, 5].

The first VFC was introduced in 2011 at Glasgow Royal Infirmary, in the hope that it would provide a standardised pathway for future acute orthopaedic care, to improve clinical efficacy and patient experiences [2, 3]. Following this, in 2013, new guidelines under the British Orthopaedic Association Standards for Trauma (BOAST) were released which stated that ‘following acute traumatic orthopaedic injury, patients should be seen in a new fracture clinic within 72 h of presentation’ to ensure standardisation of treatment in the UK [6]. This has mounted pressure onto fracture clinics to adhere to these guidelines [6]. In addition, in the NHS, there are approximately 2 million emergency department attendees every month, a 30% rise since 2004 with 30% of these musculoskeletal injuries [4]. The increasing demands on the fracture clinic precipitated by these factors have demonstrated the need for a new model to replace the FTF fracture clinic [4]. In 2020 the COVID-19 pandemic forced this change from a FTF fracture clinic into a VFC to keep patients and staff safe [7]. Since, VFCs have now largely remained in the UK [6].

In this paper, we aim to assess the clinical efficacy of VFCs and the satisfaction of patients following care provided by VFCs through systematic review of the literature. The last systematic reviews that assessed the efficacy and usefulness of VFCs were published in 2020, and we will review the current data now post COVID-19 pandemic [6, 7]. As there is now widespread use of VFCs in the UK, we hope to gain a more accurate insight into the efficacy of VFCs over a longer period of time to assess whether there is evidence that the VFC model should be the future of acute traumatic orthopaedic (ATO) care in the UK.

## Methods

A systematic review was conducted using the Preferred Reporting Items for Systematic Reviews and Meta-Analyses (PRISMA) guidelines.

### Eligibility criteria

This systematic review included retrospective cohort studies, case series and interrupted time series (ITS), prospective and cross-sectional cohort studies, closed loop audits and service evaluations. All included studies took place within the UK. For inclusion, studies had to include patients managed through the VFC as well as report satisfactory outcomes relating to the clinical efficacy or patient satisfaction of the VFC. Studies were excluded if they focused solely on paediatric patients, did not report sufficient outcomes or only reported patients managed through the FTF fracture clinic without VFC comparison.

### Primary outcomes

The primary outcomes assessed in this paper were the clinical efficacy and patient satisfaction of the VFC.

The clinical efficacy of the VFC relates to its ability to able to achieve its intended outcomes. We measured clinical efficacy using several categories: VFC discharge rates, adherence to BOAST 72-h guidelines, missed injuries, inappropriate referrals/radiographs, reattendances, and the number of FTF fracture clinic follow ups following VFC review. A direct discharge (DD) from the VFC represents a patient that has been reviewed in the VFC and discharged without further follow up appointments. We defined a reattendance as a patient reattending to the VFC following an initial discharge.

Patient satisfaction was measured through patient questionnaires. Patients who completed the questionnaires were managed through the VFC within the allocated time period for each study. These questionnaires focused on overall satisfaction and preference for either the VFC or the FTF fracture clinic.

### Secondary outcomes

The secondary outcomes of clinical efficacy were patient reported outcomes measures (PROMs) and any comparisons between VFCs and FTF fracture clinics.

Several PROMs scales were used including the EuroQoL Group 5D-5L (EQ-5D-5L) score, Quick Disabilities of the Arm, Shoulder and Hand (Quick DASH) score and the Foot and Ankle Ability Measure (FAAM). The EQ-5D-5L is comprised of the EQ-5D descriptive system and the EQ-VAS score [8]. The EQ-5D questions patients mobility, self-care, routine, pain and anxiety/depression whereas the EQ-VAS scores a patient’s health from 1 to 100 [8]. A score of 1 meaning worst possible health and 100 relating to best possible health [8]. The QuickDASH score is a questionnaire consisting of 11 questions, on a five-point Likert scale, that measures disability in the upper limbs, with the score ranging from 0 to 100 [9]. A score of 0 meaning no disability and a score of 100 relating to the highest level of disability. The FAAM consists of 29 questions, on a five-point Likert scale, that measures function in patients with foot and ankle injuries, where a higher percentage score relates to a higher level of function [10].

Some questionnaires in studies introduced secondary measures of patient satisfaction including recommendation rates, the helpfulness of advice provided and dissatisfaction rates. Additionally, Sharma et al. used specific scoring tools in their questionnaire [8]. Net promoter score (NPS) relates to the recommendation rate of the VFC [8]. Customer effort score (CES) takes into account the effort that a patient has to go through when being managed through the VFC [8]. Customer satisfaction score (CSS) measures the satisfaction of patients with the VFC service [8].

### Literature search strategy

Two reviewers (JWJ and AHK) independently conducted a systematic review of the PubMed and MEDLINE databases as well as the Cochrane library to search the literature relating to our PICO question ‘In patients diagnosed with bone fractures in the UK, how clinically efficacious is the virtual fracture clinic in managing these patients and how satisfied are they with their care?’.

Our search strategy used key words linked by Boolean operators. These terms were ‘virtual fracture clinic’ OR ‘VFC’ OR ‘telecommunication fracture clinic’ OR ‘virtual trauma clinic’ OR ‘remote fracture management’ OR ‘telemedicine fracture clinic’ OR ‘telehealth orthopaedic clinic’ OR ‘remote orthopaedic consultation’ OR ‘virtual bone clinic’ OR ‘telemedicine trauma clinic’ OR ‘remote trauma follow-up’ AND ‘NHS’.

This search did not incorporate any language restrictions but included a publication date restriction. All databases and registers were searched from 2011, to capture all available data post VFC introduction, up to 30th April 2025, when the final search was completed.

### Study selection

Two reviewers (JWJ and AHK) independently screened the titles and abstracts of all studies generated by the literature search. Following this, the full texts of appropriate identified studies were reviewed independently by JWJ and AHK and they included any studies that satisfied the eligibility criteria. On independent review, AP confirmed that there was no discrepancy between the selection of studies by JWJ and AHK for inclusion. Therefore, there was no need for any intervention.

### Data extraction and management

Data extracted from our included studies was formatted in a spreadsheet in Microsoft Excel. This spreadsheet contained the following characteristics and demographics from our included studies: primary author, study timescale, year of publication, journal, type of study, level of evidence and the population of patients assessed. This spreadsheet also documented study sample sizes and all primary and secondary outcome data. Two reviewers (JWJ and AHK) collectively extracted this data. Additionally, they discussed and resolved any inconsistencies that arose.

### Data synthesis

Data analysis was performed by two authors (JWJ and AHK). Considerable heterogeneity was identified between our studies. Therefore, no formal meta-analysis was appropriate. Instead, descriptive statistical analysis, in the form of means and ranges, was performed for our primary and secondary outcomes.

### Quality assessment

Two reviewers (JWJ and AHK) performed simultaneous independent assessment of the quality of our included studies by evaluating their risk of bias. Any variability in bias assessment was discussed and reevaluated independently. If a non-randomised observational study was identified the Newcastle Ottawa Scale (NOS) was used and if a closed loop audit was identified the JBI Critical Appraisal Checklist for Quasi-experimental studies was used to assess bias.

## Results

### Literature search results

**Fig. 1:**
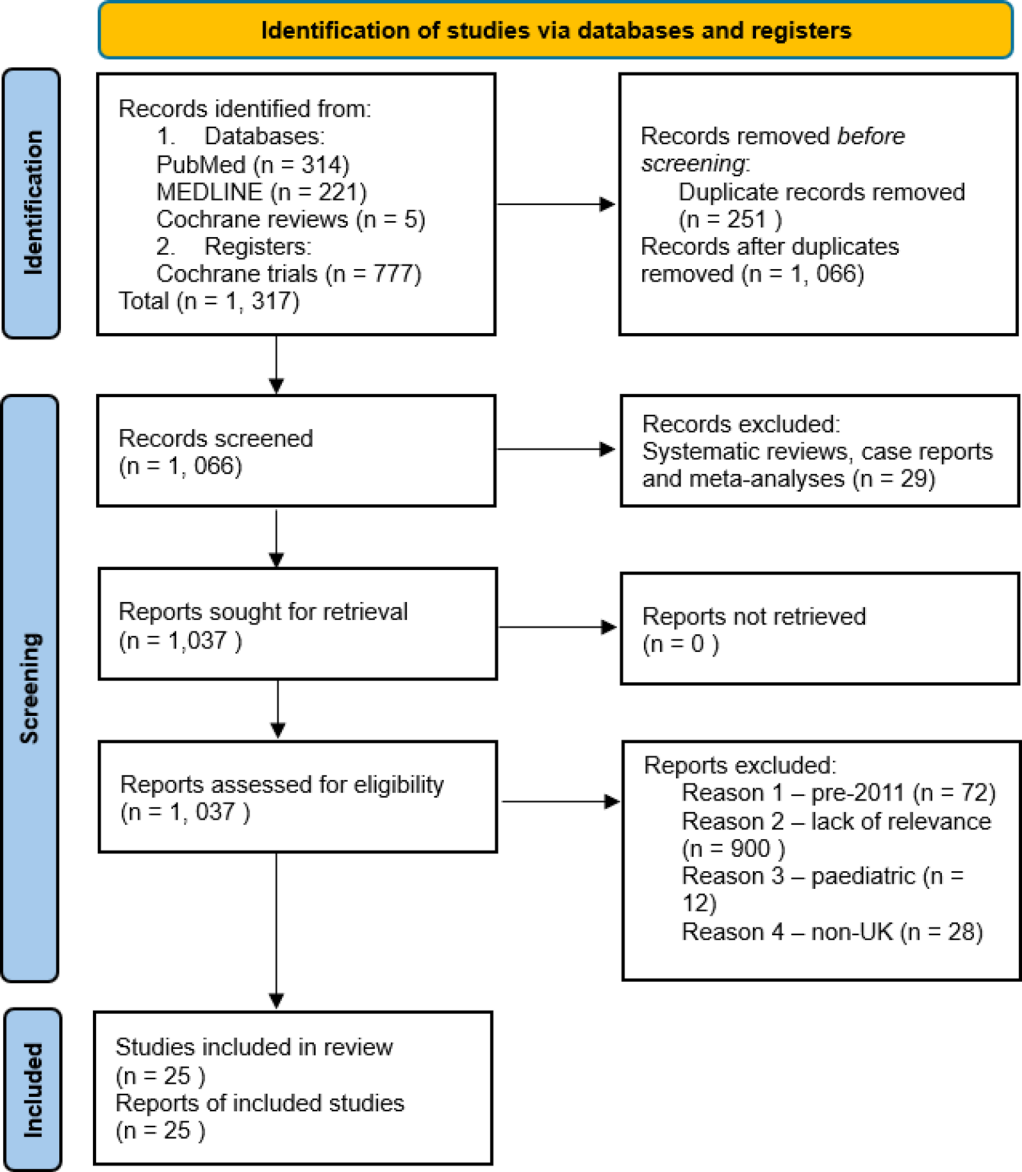
PRISMA flowchart of search strategy. This flowchart depicts the steps taken in screening studies to be included in this systematic review as well as the figures at each stage of the process. Adapted figure from PRISMA 2020 flow diagram—PRISMA statement [11]

Searching the literature using our search strategy yielded 1,317 results. After two reviewers (JWJ and AHK) systematically screened these studies using the PRISMA guidelines, a total of 25 studies were identified that satisfied our eligibility criteria (Fig. 1).

### Study characteristics and demographics

Out of our 25 included studies, 4 were exclusively focused on adult patients [8, 12, 19, 24]. Seven studies assessed a mixed population consisting of both adult and paediatric cases [3, 5, 15, 17, 18, 22, 31]. Fourteen studies did not specify their exact population [13, 14, 16, 20, 21, 23, 25–30, 32, 33]. Fifteen studies assessed mixed fracture types [3, 5, 8, 12–16, 22, 24, 25, 28–30, 33]. Two studies focused on 5th metatarsal base fractures [18, 26]. Eight studies focused solely on specific types of fractures, these included clavicle, hand and wrist, hand, elbow hand and foot, scaphoid, Weber B ankle or toe [17, 19–21, 23, 27, 31, 32]. We reviewed a range of papers pre, intra and post the COVID-19 pandemic peak in 2020. Eight studies took place during this period [8, 12, 18–20, 24, 25, 30]. Eleven studies took place before this period [13, 16, 21, 23, 26–29, 31–33]. Six took place after this period [3, 5, 14, 15, 17, 22]. The mean study timescale was 9.5 months, which demonstrates that our studies had sufficient time to gather appropriate amounts of data [3, 5, 8, 12–33] (Tables 1 and 2).

**Table 1:** Characteristics of included studies

Author	Study timescale	Year	Journal	Study type	Level of evidence
Sharma et al. [8]	2 weeks–23rd March to 5th April 2020	2021	Cureus	Retrospective cohort study	Level III
Zhan et al. [12]	Pre-pandemic: 2 weeks–6th May 2019 to 19th May 2019 Post pandemic: 2 weeks–4th May 2020 to 17th May 2020	2024	Injury	Cross sectional cohort study	Level III
Thelwall [13]	2 months–July and Aug 2019	2021	Int J Orth Traum Nurs	Service evaluation	Level IV
Smitheman et al. [14]	1 month–Dec 2022	2025	J Clin Orth & Traum	Retrospective cohort study	Level IV
Bakhiet et al. [15]	7 months–9th Sept 2022 to 31st March 2023	2023	Cureus	Retrospective cohort study	Level IV
Dey et al. [16]	17 months–Sept 2017 to Feb 2020	2022	Ann R Coll Surg Engl	Retrospective cohort study	Level IV
Bin Sahl et al. [17]	27 months–1st Jan 2022 to 21st April 2024	2024	Cureus	Retrospective cohort study	Level IV
Toner et al. [18]	14 months–Feb 2019 to April 2020	2024	Eur J Orth Surg & T	Retrospective cohort study	Level IV
Morcos et al. [19]	2 months–April and May 2020	2024	HAND	Retrospective cohort study	Level III
Bhavanasi et al. [20]	4 months–8th July 2020 to 11th Nov 2020	2024	Foot and ankle Orth	Retrospective cohort study	Level IV
Mackenzie et al. [21]	46 months–Feb 2014 to Dec 2017	2022	Injury	Retrospective cohort study	Level IV
Williams et al. [22]	Phase 1: 2 months–Aug to Sept 2021, Phase 2: 3 months–Jan to March 2022, Phase 3: 2 months Aug to Sept 2022	2023	Injury	Service evaluation	Level III
Dutta et al. [23]	Pre-pathway: 4 months–Feb to May 2017, Post-pathway: 3 months–May to July 2018 VFC introduced in Feb 2018	2021	Ann R Coll Surg Engl	Retrospective cohort study	Level III
Jain et al. [24]	9 months–May 2020 to Jan 2021	2024	Cureus	Cross-sectional cohort study	Level IV
Dunkerley et al. [25]	2 weeks–14th to 28th April 2020	2020	Int Orth	Prospective cohort study	Level II
Galloway et al. [26]	12 months–Jan 2019 to Dec 2019	2023	Injury	Retrospective case series	Level IV
Bellringer et al. [27]	24 months–Sept 2013 to Sept 2015	2017	Injury	Retrospective cohort study	Level III
McKirdy et al. [28]	35 months–May 2013 to April 2016	2017	BJR	Retrospective ITS	Level III
Das et al. [3]	12 months–17th March 2021 to 16th March 2022	2024	Cureus	Retrospective cohort study	Level IV
Sephton et al. [29]	32 months–July 2017 to March 2020	2021	Eur J T Emerg Surg	Retrospective cohort study	Level III
Dunkerley et al. [30]	Audit: 1 month–1st April to 28th April 2020 Re-audit: 2 weeks–18th May to 29th May 2020	2020	Injury	Closed loop audit	Level III
Bhattacharyya et al. [31]	12 months–Oct 2011 to Sept 2012	2017	Injury	Retrospective cohort study	Level IV
Little et al. [32]	Pre-study comparison: 3 months–Sept to Dec 2016 and Study: 19 months–Jan 2017 to Oct 2018	2020	JHS (E)	Prospective cohort study	Level IV
Nagy et al. [5]	5 months–Jan to May 2022	2024	Cureus	Prospective cohort study	Level II
Holgate et al. [33]	1st cycle: 1 month–May 2014 2nd cycle: 1 month–Jun 2015 VFC went live in Oct 2014	2017	Ann R Coll Surg Engl	Closed loop audit	Level III

Characteristics include author, study timescale, year, journal, study type, and level of evidence.

Oxford Centre for Evidence-Based Medicine: Levels of evidence was used to assess each study.

**Table 2:** Demographics and assessed outcomes of included studies

Author	CE sample size	Population	Injuries assessed	CE	PS	PROMs
Sharma et al. [8]	49	Adults	Mixed	Yes	Yes	Yes
Zhan et al. [12]	3, 877	Adults	Mixed	Yes	Yes	No
Thelwall [13]	141 + 557	N.S	Mixed	Yes	Yes	No
Smitheman et al. [14]	397	N.S	Mixed	Yes	No	No
Bakhiet et al. [15]	123	Mixed	Mixed	Yes	No	No
Dey et al. [16]	17, 269	N.S	Mixed	Yes	No	No
Bin Sahl et al. [17]	N/A	Mixed	Toe	No	Yes	Yes
Toner et al. [18]	194 + 136	Mixed	5th metatarsal base	Yes	Yes	Yes
Morcos et al. [19]	26	Adults	Hand	Yes	Yes	Yes
Bhavanasi et al. [20]	175	N.S	Foot and ankle	Yes	No	No
Mackenzie et al. [21]	6, 688	N.S	Elbow, hand and foot	Yes	No	No
Williams et al. [22]	4, 810	Mixed	Mixed	Yes	Yes	No
Dutta et al. [23]	176 + 111	N.S	Scaphoid	Yes	No	No
Jain et al. [24]	522	Adults	Mixed	Yes	Yes	No
Dunkerley et al. [25]	154	N.S	Mixed	Yes	Yes	No
Galloway et al. [26]	126	N.S	5th metatarsal base	Yes	Yes	Yes
Bellringer et al. [27]	415	N.S	Weber B ankle	Yes	No	No
McKirdy et al. [28]	5, 958	N.S	Mixed	Yes	No	No
Das et al. [3]	4, 819	Mixed	Mixed	Yes	No	No
Sephton et al. [29]	12, 266	N.S	Mixed	Yes	No	No
Dunkerley et al. [30]	197 + 133	N.S	Mixed	Yes	No	No
Bhattacharyya et al. [31]	138	Mixed	Clavicle	Yes	Yes	Yes
Little et al. [32]	3, 709	N.S	Hand and wrist	Yes	Yes	Yes
Nagy et al. [5]	150	Mixed	Mixed	Yes	Yes	No
Holgate et al. [33]	51	N.S	Mixed	Yes	No	No

Demographics include clinical efficacy (CE) sample sizes, population and injuries assessed. Mixed population = adults + paediatric. N.S = does not specify. PS = patient satisfaction.

This paper reviewed the clinical efficacy data of 24 studies from a very large sample size of 63,367 patients [3, 5, 8, 12–16, 18–23, 25–33]. The numbers of patients from our studies ranged from 26 to 17,269 with a mean of 2,640 patients [3, 5, 8, 12–16, 18–23, 25–33].

### VFC discharge rates and BOAST guidelines

Eighteen studies expressed data on VFC discharge rates [3, 5, 12–14, 16, 18, 20–23, 25, 26, 28–32]. Fourteen focused on DDs [3, 5, 12–14, 16, 18, 20–23, 26, 31, 32]. VFC DD rates in our studies ranged from 5 to 99.2% [3, 5, 12–14, 16, 18, 20–23, 26, 31, 32]. Smitheman et al. had a 5% DD rate, a sample size of 397 and focused on mixed fracture types but only took place over 1 month [14]. Galloway et al. had a 99.2% DD rate and an acceptable sample size of 126 but only focused on 5th metatarsal base fractures, a cohort of patients that may be more inclined for DD than more complex fracture types [26]. Zhan et al. displayed an increase from 30 to 40% in VFC DD rate in 3877 patients, in their study during the COVID-19 pandemic, on mixed fracture types [12]. Dutta et al. showed a similar trend in scaphoid fractures after VFC implementation in 2018, with an increase in DD from 83.3 to 87.2%, in patient populations of 176 and 111 respectively [23]. Dutta et al. also showed an increase in the number of patients managed through the VFC from 6.8 to 42.3% post VFC implementation [23]. Overall, VFCs had consistently high DD rates with a mean rate of 47.7% from these 18 studies [3, 5, 12–14, 16, 18, 20–23, 25, 26, 28–32] (Table 3).

**Table 3:** Clinical efficacy of the VFC–VFC discharge rates and BOAST guidelines

Author	VFC discharge rates	BOAST guidelines
Zhan et al. [12]	During the pandemic DD % from VFC without FTF review increased from 30 to 46% with low intervention rates following DD	
Thelwall [13]	Aug 2019—141 (31%) of patients DD	Jul 2019–80% of patients seen within VFC within 72 h
Smitheman et al. [14]	18(5%) DD	
Bakhiet et al. [15]		94.5% VFC consultations per month within 72 h an increase of 7.3% over study period
Dey et al. [16]	9, 908 (57.4%) DD rate 9, 167 (92.52%) of these virtual discharges managed successfully	Pre- VFC implementation: 6% patients seen within 72 h, mean waiting time of 10.7 days Post-VFC implementation: 100% of patients reviewed within 72 h, mean waiting time of 1.2 days
Toner et al. [18]	80 (41.2%) DD	
Bhavanasi et al. [20]	85 (48.6%) DD and managed via VFC	
Mackenzie et al. [21]	4810 (71.9%) DD	
Williams et al. [22]	1140 (24%) DD before phase 1 and after phase 2 The DD rate increased to 36% after phase 3	From phase 3 onwards, 65% of patients reviewed within 72 h
Dutta et al. [23]	Pre-VFC pathway 176 patients: 12 (6.8%) VFC managed and 10 (83.3%) DD Post-VFC pathway 111 patients: 47(42.3%) VFC managed and 41 (87.2%) DD	
Dunkerley et al. [25]	108 (71%) given patient initiated follow up discharge	
Galloway et al. [26]	125 (99.2%) DD	Median time 2 days from ED to VFC within 72 h Comparably regular pathway takes 14 days from ED to VFC
Bellringer et al. [27]		Mean average days from injury to radiograph was 3 days
Mckirdy et al. [28]	Monthly VFC discharge rates 32.9%	Pre-VFC intervention 5.1% patients had an appointment within 72 h Post-VFC intervention 46.4%
Das et al. [3]	1704 (35.4%) DD	
Sephton et al. [29]	393 (13.1%) out of 3, 308 paper based, 1975 (20.8%) out of 9,228 electronic based discharged from VFC	87.6% paper and 94.9% electronic—time from referral to assessment within 72 h
Dunkerley et al. [30]	Audit: 197 (88%) patients seen in VFC, all audit standards above 90% across 4 standards, 97% of patients were either virtually assessed in follow up or had documented reason for attendance for follow up	BOAST guidelines (7–10) met
	Re-audit: 133 (90%) reviewed in VFC, 100% of patients were either virtually assessed in follow up or had documented reason for attendance for follow up, used the same audit standards	BOAST guidelines (7–10) met
Bhattacharyya et al. [31]	62 (45%) DD	
Little et al. [32]	968 (26%) DD	Pre-VFC implementation: patient wait time 10 days and time to treatment initiation by specialist 18 days Post-VFC implementation: 1.2 days and 11 days respectively
Nagy et al. [5]	67 (44%) DD	VFC helped hit BOAST guidelines 72 h target
Holgate et al. [33]		Pre-VFC introduction 123 patients: 7 (6%) seen in 72 h mean wait time 10.5 days, post-VFC introduction 51 patients: 100% seen within 72 h, mean wait time 1.3 days

This table details VFC discharge rates and whether VFCs in our included studies met BOAST guidelines (in particular BOAST 72-h guidelines). This table also displays comparisons in BOAST 72-h guideline compliance before and after VFC implementation in appropriate studies

Three studies reported high total or patient initiated follow up VFC discharge rates [25, 28, 29]. Sephton et al. reported VFC discharge rates of 33.9% over their 32-month study out of a total sample size of 12,266 in mixed fracture types [29]. Similarly, McKirdy et al. reported a monthly average VFC discharge rate of 32.9% over their 35-month study out of a total sample size of 5,958 in mixed fracture types [28]. Whereas, Dunkerley et al. displayed a 71% patient initiated follow up VFC discharge rate in their 154 patients with mixed fracture types [25].

Two studies described high numbers of successful patient management following VFC discharge [12, 16]. Dey et al. had the largest sample size, 17,269, with mixed fracture types and reported a VFC DD rate of 57.4% over 17 months, with 92.5% of these being managed successfully [16]. This is further supported by Zhan et al., who described low intervention rates following VFC DD [12].

Twelve studies commented on the ability of VFCs to meet the BOAST guidelines, in particular BOAST 72-h guidelines [5, 13, 15, 16, 22, 26–30, 32, 33]. These studies displayed that there is a high percentage of patients reviewed within 72-h by the VFC following their initial presentation. These results range from 46.4 to 100%, with a mean of 83.6% [5, 11, 13, 14, 20, 24–28, 30, 31]. McKirdy et al. had a 46.4% adherence rate [28]. Whereas two studies, Dey et al. and Holgate et al. both with mixed fracture types, reported 100% adherence to BOAST 72-h guidelines, although Holgate et al. only reported data for 51 patients [16, 33]. Bakhiet et al. also reported an increase of 7.3% in adherence rates to BOAST 72-h guidelines in their study of 123 patients with mixed fracture types across 2022 to 2023 [15].

Five studies reported data on the wait times for patients from their initial presentation until VFC review [16, 26, 27, 32, 33]. These studies correlated with our high adherence rates to BOAST 72-h guidelines, with all results ≤ 72 h. These results range from 1.2 days to 3 days mean wait time, with an overall mean of 1.7 days [16, 26, 27, 32, 33]. Dey et al., Little et al. and Holgate et al. had a mean wait time of 1.2 days [16, 32, 33]. Whilst, Bellringer et al. had a mean wait time of 3 days in 415 patients, however they only focused on Weber Type B ankle fractures [27]. Additionally, Galloway et al. expressed a median wait time of 2 days [26].

Comparably, 5 studies detailed the differences between VFCs and the FTF fracture clinics in their ability to meet BOAST 72-h guidelines [16, 26, 28, 32, 33]. These studies showed that VFCs are much more efficacious than FTF fracture clinics in meeting BOAST 72-h guidelines as well as reducing patient wait times. Between Dey et al., Mckirdy et al. and Holgate et al. the mean rate of FTF fracture clinics meeting BOAST 72-h guidelines was only 5.7%, a 77.9% decrease from mean VFC adherence rates [16, 28, 33]. Also, between Dey et al., Little et al. and Holgate et al. the mean patient wait time of FTF fracture clinics from presentation until review was 10.4 days, a 9.2 day increase in mean patient wait times from VFCs [16, 32, 33]. Additionally, Galloway et al. displayed a median patient wait time of 14 days for FTF fracture clinics [26].

Little et al. reported a solitary result on the mean time to treatment initiation by a specialist if required, and this was 11 days out of a total sample size of 3,709 in hand and wrist fractures [32]. An audit by Dunkerley et al. used 4 BOAST guidelines as their audit standards [30]. They showed that during the COVID-19 pandemic peak, with VFCs prominent, all of these guidelines were met in both their audit and re-audit of mixed fracture types with 197 and 133 patients respectively [30, 34].

### Missed injuries, inappropriate referrals/radiographs and reattendances

Only 2 studies commented on missed injuries by VFCs [12, 32]. Albeit these studies showed that the VFC has extremely low rates of missed injuries. Zhan et al. had only 31 out of 3,877 (0.8%) missed injuries whilst Little et al. had only 2 missed injuries out of 968 (0.2%) [12, 32]. The two missed injuries reported in Little et al. were identified on VFC review and as a result these patients were recalled for in person review [32] (Table 4).

**Table 4:** Clinical efficacy of the VFC—Missed injuries, inappropriate referrals/radiographs and reattendances

Author	Missed injuries and inappropriate referrals/radiographs	Reattendances
Zhan et al. [12]	31 (0.8%) missed injuries	262 (6.8%) unexplained reattendances
Thelwall [13]		Jul 2019—9 out of 557 (1.6%) reattended
Smitheman et al. [14]	3 (1%) rejected referrals	3 (1%) called back following discharge
Dey et al. [16]	961 (5.6%) inappropriate referrals	742 (7.5%) reattendances
Morcos et al. [19]		No planned reattendances within first year of injury
Mackenzie et al. [21]		298 of 4810 (6%) reattended
Williams et al. [22]		116 (10%) reattended due to symptoms
Galloway et al. [26]		@@12 (9.5%) reattended, 9 for pain
Sephton et al. [29]	Rejected referrals: 26 (0.9%) paper, 280 (2.9%) electronic	
Dunkerley et al. [30]	0.4% of patients had inappropriate radiographs	
	0.7% of patients had inappropriate radiographs	
Bhattacharyya et al. [31]		1 reattendance for sling adjustment (1.6%)
Little et al. [32]	2 patients with missed injuries from MIU were recalled via the VFC	

This table details the numbers of missed injuries and inappropriate referrals or radiographs through the VFC in our included studies. Additionally, this table includes reattendances and the reasons for reattendance if available

Three studies detailed inappropriate referrals with Dey et al. identifying 961 (5.6%) of their referrals to be inappropriate and Smitheman et al. and Sephton et al. reporting < 3% of their referrals being rejected [14, 16, 29]. One study, Dunkerley et al., detailed data numbers of inappropriate radiographs, with 0.4% in their audit and 0.7% in their re-audit [30]. These results display VFCs have both low levels of inappropriate referrals and inappropriate radiography orders.

Nine studies displayed that there are low reattendance rates following discharge from the VFC [12–14, 16, 19, 21, 22, 26, 31]. There was minimal variability between reattendance measurements in these studies and therefore many comparable results were extracted. The range of reattendance rates from these studies was 1% to 10%, with a 4.9% mean rate of reattendance [12–14, 16, 19, 21, 22, 26, 31]. Morcos et al. reported no re-attendances within the first year following discharge from the VFC in their patients, however the sample size was small with only 26 patients and they only focused on hand fractures over 2 months [19]. Smitheman et al. and Williams et al. reported a 1% and 10% reattendance rate respectively, both with appropriate sample sizes and mixed fracture types [14, 22]. Most studies did not state the reason for reattendance however, Galloway et al. and Williams et al. explained that their reattendances were due to ongoing pain and symptoms [22, 26].

### FTF clinic appointments and FTF fracture clinics vs VFCs comparison

Three studies reported a significant reduction in the number of FTF clinical appointments when VFCs were introduced [23, 28, 30]. Dutta et al. and McKirdy et al. detailed a reduction in FTF clinical appointments from 164 to 64 and 461 to 140 respectively [23, 28]. In addition, Dunkerley et al. stated that in their initial audit, implementation of a VFC instead of a FTF clinic could have saved 22 FTF appointments and 13 X-rays [30]. Furthermore, McKirdy et al. reported that consultants spent longer with their patients in FTF clinic appointments following VFC implementation [28]. On average, the time they spent with their patients increased from 13 min 44 s to 14 min 53 s [28]. This displays that VFCs can have a positive impact on the care delivered in FTF clinical appointments as a result of the reduction in the number of appointments (Table 5).

**Table 5:** Clinical efficacy of the VFC–FTF clinic appointments and VFC comparison

Author	FTF clinic review appointments	FTF vs VFC
Sharma et al. [8]		Greater satisfaction and outcomes when at least one FTF appointment in combination with VFC rather than solely VFC
Smitheman et al. [14]	177 (45%) FTF clinical review	
Dey et al. [16]	6, 400 (37.1%) FTF clinical review	
Toner et al. [18]	114 (58.8%) offered at least one FTF follow up	
Mackenzie et al. [21]	951 (14.2%) offered FTF clinical review 47 (5%) requested an in person review	
Dutta et al. [23]	64 (57.7%) FTF clinic appointments	VFC pathway implementation reduced no. FTF clinic appointments required from 164 (93.2%) to 64 (57.7%)
Jain et al. [24]	252 (48%) required FTF physical exam follow up	
Dunkerley et al. [25]	19 (12%) seen in FTF clinic with 27 (17%) requiring a virtual follow up	
Mckirdy et al. [28]		Pre-VFC implementation: number of FTF clinic appointments 461, mean time per consultation in FTF clinics 13 min 44 s Post-VFC implementation: 140.4 and 14 min 53 s
Dunkerley et al. [30]	Audit: 18 (8%) seen FTF with prior approval	If VFC rather than FTF could have saved 22 appointments and 13 X ray’s
	Re-audit: 14 (10%) seen FTF with prior approval	
Bhattacharyya et al. [31]	76 (55%) seen in FTF clinic after VFC review	
Little et al. [32]	2086 (56%) offered FTF clinic	
Nagy et al. [5]	67 (44%) of patients booked FTF appointment	19 out of 67 patients (28%) reported difficulties getting to FTF appointment 88% of patients understood their management over the phone Reduction in non-attends: 82 to 6 Reduction in complaints regarding appointments: 72 to 22
Holgate et al. [33]	51 (87.9%) patients seen in FTF clinic post-VFC introduction	

This table details the number of FTF clinic appointments following VFC review, and any further comparisons not previously explored between data from VFCs and FTF fracture clinics in our included studies

Twelve studies reported data on the numbers of patients who were seen in or offered a FTF clinical appointment following VFC review [5, 14, 16, 18, 21, 23–25, 30–33]. The range of FTF clinical review rates were 8–87.9%, with an average of 42.4% [5, 14, 16, 18, 21, 23–25, 30–33]. This shows that there was a high recall rate for in person clinical appointments for patients following VFC review. Dunkerley et al. had an 8% FTF clinical review rate in their first audit, and a 10% rate in their re-audit [30]. Holgate et al. reported an 87.9% FTF clinical review rate in their 2nd audit in June 2015 however this result is limited by their small sample size [33]. Jain et al. reported that in total 65.3% of their 522 patients were required to reattend for FTF review, in their 9-month study of mixed fracture types, with 48% of these for physical examination [24]. Mackenzie et al. stated that 5% of their patients in their 45 month elbow, hand and foot fracture study, in a total sample size of 6,668, requested an in person review [21].

Two studies documented comparisons between FTF fracture clinics and VFCs that have not yet been explored in this paper [5, 8].

Sharma et al., in their 2-week study of mixed fracture types concluded that there is greater satisfaction and outcomes for patients when they have at least one FTF appointment in combination with a VFC rather than only being managed virtually [8]. Although, this only evaluated outcomes in their small sample size of 49 patients [8].

Nagy et al. conducted a 5-month study of mixed fracture types and measured complaints, accessibility, management plan understanding and non-attendance rates before and after VFC implementation in a total sample size of 150 patients [5]. They showed that complaints and non-attends decreased from 72 to 22 and 82 to 6 respectively, after the VFC was introduced [5]. They also reported that 28% of patients had difficulties getting to their FTF appointment and 142 out of 150 patients (88%) understood their management over the phone [5].

### PROMs

PROMs data was reviewed from 6 studies, with a total of 1,313 patients, a range from 26 to 984 patients and a mean of 218 patients [8, 17–19, 31, 32]. This can be further subdivided into a total of 247 patients reviewed for the EQ-5D-5L score, 1,072 patients for the QuickDASH score and 192 patients for the FAAM [8, 17–19, 31, 32] (Table 6).

**Table 6:** PROMs following VFC review

Author	Sample size	EQ-5D-5L	QuickDASH	FAAM
Sharma et al. [8]	49	EQ-VAS mean EQ-VAS of 73.678 for VFCs		
Bin Sahl et al. [17]	56			100% median score
Toner *et al* [16]	136	EQ-5D-5L median score 0.95, EQ-VAS median score 85		95% median score
Morcos et al. [19]	26		Median score 2.3, 5 had higher scores	
Bhattacharyya et al. [31]	62	EQ-VAS mean score 78.1	Mean score 16.1	
Little et al. [32]	984		All scores good or excellent	

This table details the sample sizes for PROMs data in our included studies as well as the EQ-5D-5L, Quick DASH and FAAM scores if reported

Three studies used the EQ-5D-5L system to measure PROMs [8, 18, 31]. Bhattacharya et al. and Sharma et al. reported mean EQ-VAS scores of 73.678 and 78.1, while Toner et al. found a median EQ-VAS score of 85 [8, 31]. These results show that patients have high levels of health following management through the VFC, especially in Sharma et al. which reported on mixed fracture types [8]. Three studies used the QuickDASH score to measure PROMs [19, 31, 32]. Morcos et al. found a median score of 2.3, whilst Bhattacharya found a mean score of 16.1 and Little et al. presented data qualitatively with all having good or excellent scores [19, 31, 32]. These results signify that patients have low levels of upper limb disability following management through the VFC. Two studies used the FAAM score to measure PROMs [17, 18]. Bin Sahl et al. and Toner et al. reported 100% and 95% median scores respectively [17, 18]. These figures displays that patients have high levels of function following toe and 5th metatarsal base fractures managed through the VFC.

### Patient satisfaction

Thirteen studies reported data on the satisfaction of patients following management through the VFC with a large sample size of 3,758 patients, a range from 26 to 1,527 patients and a mean of 289 patients [5, 8, 12, 13, 17–19, 22, 24–26, 31, 32]. Due to the variation between questionnaires, we grouped all measurements of satisfaction together into ‘overall satisfaction’. Nine studies fell under this category and displayed high levels of patient satisfaction following VFC management [5, 12, 17–19, 24–26, 31, 32] (Table 7).

**Table 7:** Patient satisfaction following VFC review

Author	Sample size	Questionnaire	Result
Sharma et al. [8]	49	Validated questionnaire—NPS, CES and CSS on scale of 1–10	NPS = 7.29, CES = 7.2, CSS = 7.55
Zhan et al. [12]	1, 527	Questionnaire: 1. Satisfaction 2. Preference 3 Dissatisfaction reasons	Pre-lockdown: satisfaction 80%, dissatisfaction 20%, post-lockdown: satisfaction 76%, dissatisfaction 24% 687 (45%) prefer VFC, 492 (32.2%) prefer FTF and 348 (22.8%) no preference Dissatisfaction—injury missed at VFC review, change in management or unplanned reattendance, not receiving call, need to use or unaware of helpline
Thelwall [13]	41	Questionnaire: 1. Recommendation rate 2. Preference	88% recommendation rate for service 17% of patients in 2019 would have preferred FTF compared to 9% in 2015
Bin Sahl et al. [17]	52	Questionnaire: 1. Experience 2. Helpful advice 3. Complaints	48 patients (92.9%) + ve experiences 51 (98.2%) advice helpful 50% complaints due to insufficient communication
Toner et al. [18]	135	Satisfaction questionnaire	126 patients (93.3%) satisfied with current level of function and recovery
Morcos et al. [19]	26	Satisfaction questionnaire	73.1% satisfied or very satisfied with treatment and outcomes
Williams et al. [22]	87	Satisfaction questionnaire 1–10	7.3 mean for VFC whereas 8.7 for FTF
Jain et al. [24]	522	Questionnaire: 1. Satisfaction 2. Preference	77.9% satisfaction from VFC 22% preferred FTF
Dunkerley et al. [25]	100	Questionnaire: 1. Satisfaction 2. Preference 1–5	Average satisfaction 4.8/5 (96%) 11 preferred FTF and of these patients satisfaction still 4.4/5 (88%)
Galloway et al. [26]	118	16 question satisfaction questionnaire	115 (97.5%) responded ‘yes’ were happy with injury management on VFC, 1 (0.8%) responded no, in 2 patients the question was not asked
Bhattacharyya et al. [31]	44	4 question satisfaction questionnaire	Overall satisfaction of clinical outcome 40 (91%) patients, 4 dissatisfied (9%)—had to use helpline to obtain further info
Little et al. [32]	907	Satisfaction questionnaire	468 (52%) highly satisfied, 433 (48%) satisfied and 6 (0.7%) dissatisfied Out of dissatisfied patients: 3 preferred to see a doctor, 2 lack of relevant info, 1 difficult to get follow up
Nagy et al. [5]	150	Satisfaction questionnaire	112 (74.6%) of patients satisfied with VFC, 14 (9.3%) dissatisfied Dissatisfied patients reported call too quick, struggled to hear or keen for further FTF assessment

This table details the sample size, type of questionnaire and results of patient satisfaction from our included studies

The range of satisfaction from these studies was 73.1% to 97.5% with a mean satisfaction of 85.4% [5, 12, 17–19, 24–26, 31, 32]. Morcos et al. had the lowest satisfaction rate of 73.1%, however this result is bound by its small sample size of 26 patients as well as only focusing on hand fractures [19]. Galloway et al. had the highest satisfaction rate of 97.5% in a population of 118 patients, however it only focused on 5th metatarsal base fractures [26]. Williams et al. and Dunkerley et al. quantified the level of satisfaction in their patients with high mean satisfaction scores of 7.3/10 and 4.8/5 respectively [22, 25]. Sharma et al. did not use a typical satisfaction questionnaire but instead used NPS, CES and CSS [8]. Their NPS score was 7.29 which correlates with Thelwall’s high recommendation rate of 88% [8, 13]. Their CES and CSS scores were high as well; 7.2 and 7.55 respectively [8].

Five studies assessed whether patients preferred to be treated using VFCs or FTF clinics [12, 13, 22, 24, 25]. Overall, the data showed that currently patients prefer the VFC to the FTF fracture clinic. Zhan et al. and Jain et al. both focused on mixed fracture types and had large sample sizes of 1,527 and 522 respectively [12, 24]. These studies found a 45% and 77.9% patient preference for the VFC, with only 32.2% and 22% preferring FTF fracture clinics respectively [12, 24]. Dunkerley et al. also concurred with 88.9% of their patients preferring the VFC [25]. In contrast, Thelwall showed an increase in affinity for FTF fracture clinical care with a rise from 9% in 2015 to 17% in 2019 however, this was out of a small sample size of 41 patients [13, 25]. Williams et al. performed a 5-month study of mixed fracture types but did not directly compare VFC to FTF fracture clinic preference. Instead they showed that patients managed through FTF fracture clinics actually had a higher satisfaction score of 8.7/10 than VFCs with 7.3/10 [22].

Six studies displayed results on patient dissatisfaction following VFC management and these rates were low [5, 12, 17, 26, 31, 32]. From these studies, dissatisfaction rates ranged from 0.7% to 24%, with a mean of 10.6% [5, 12, 17, 26, 31, 32]. Many of the patients who were dissatisfied with their VFC experience identified problems surrounding communication [5, 12, 17, 32]. Issues raised included struggling to hear on the phone, a lack of relevant information, the call being too quick or unclear advice [5, 12, 17, 32]^.^ Alternatively, some dissatisfied patients just preferred to see a doctor or were keen for an in person assessment [5, 32].

### Quality assessment results

The NOS was used to assess the quality and risk of bias in 23 of our studies which were observational [3, 5, 8, 12–29, 31, 32]. The NOS assesses 3 key areas: selection, comparability and outcomes and scores the quality of the paper using stars [35]. A maximum of 4 stars can be achieved for selection, 2 for comparability and 3 for outcomes [35]. The summation of a papers stars for each category forms the grade of the papers quality [35]. A score of ≥ 7 up to a maximum of 9 means the paper is high quality and at a low risk of bias, a score of 4–6 means the paper is moderate quality and at a moderate risk of bias, a score of 0–3 means the paper is low quality and at a high risk of bias [36]. Out of our included studies, 9 were high quality, 14 were moderate quality and 0 were low quality, with a star range of 5 to 9 stars and an average score of 7.14 stars indicating that there is a low risk of bias amongst these studies (Table 8).

**Table 8:** NOS for observational studies

Author	Selection	Comparability	Outcome	Total stars
Sharma et al. [8]	★★★	★	★★★	7
Zhan et al. [12]	★★★★	★★	★★★	9
Thelwall [13]	★★★		★★★	6
Smitheman et al. [14]	★★★		★★★	6
Bakhiet et al. [15]	★★★		★★★	6
Dey et al. [16]	★★★		★★★	6
Bin Sahl et al. [17]	★★		★★★	5
Toner et al. [18]	★★★		★★★	6
Morcos et al. [19]	★★★★	★★	★★★	9
Bhavanasi et al. [20]	★★★		★★★	6
Mackenzie et al. [21]	★★★		★★★	6
Williams et al. [22]	★★★★	★★	★★★	9
Dutta et al. [23]	★★★★	★★	★★★	9
Jain et al. [24]	★★★		★★★	6
Dunkerley et al. [25]	★★★		★★★	6
Galloway et al. [26]	★★★		★★★	6
Bellringer et al. [27]	★★★	★	★★★	7
McKirdy et al. [28]	★★★★	★★	★★★	9
Das et al. [3]	★★★		★★★	6
Sephton et al. [29]	★★★	★	★★★	7
Bhattacharyya et al. [31]	★★★		★★★	6
Little et al. [32]	★★★★	★★	★★★	9
Nagy et al. [5]	★★★		★★	5

This table displays the results of bias assessment in our non-randomized observational studies using the NOS. Detailed in the table are the total number of stars as well as a breakdown of the star allocation

For our two remaining studies, which are both closed loop audits, we used the JBI Critical Appraisal Checklist for Quasi-experimental studies to assess the quality and risk of bias in these audits [37]. This checklist involves a series of questions in sections relating to internal and statistical validity which are answered with yes, no, unknown or N/A [37]. Finally, an overall appraisal of the study quality was decided from these answers [37]. Both audits met the checklists criteria and therefore have been included in this systematic review.

Please see Table 9 in the supplementary material for a breakdown of the quality assessment of our closed loop audits using the JBI Critical Appraisal Checklist for Quasi-experimental studies.

## Discussion

### High discharge rates and high levels of successful management following VFC review

This paper found that VFCs are very efficient in reviewing and discharging patients. DDs as well as total discharge rates and discharges with patient initiated follow up levels are high. Murphy et al. reported that VFCs in seventeen out of their 18 studies were efficient, with VFC discharges ranging from 18.2 to 100%, alongside an overall VFC discharge proportion of > 65%, which supports our findings [7]. In addition, Khan et al. reported their overall VFC discharge rate to be between 33 and 60%, which correlates with our mean VFC DD rate of 44.7% [6]. Overall, this paper indicates that VFCs are efficient in reviewing patients and are highly efficacious in providing the right care following VFC review. This is shown through the high levels of successfully managed patients that have been reviewed and discharged by the VFC.

### VFCs consistently meet BOAST guidelines and reduce patient wait times

Our results from this paper demonstrate that VFCs are extremely efficacious in providing timely care for ATO patients through their consistent ability to meet BOAST 72-h guidelines. This means that patients receive faster care through VFCs and have decreased wait times, which not only improves patient experience but may be pivotal in the event of a more complex fracture. Additionally, when compared to the compliance of FTF fracture clinics to BOAST 72-h guidelines and reducing patient wait times, VFCs are superior.

These results are reinforced by Murphy et al. who reported that all of their reviewed studies conducted their VFC follow ups within 48 h [7]. Furthermore, in alignment with the decreased patient wait times presented in this paper, 5 studies reported by Murphy et al. showed that implementation of VFCs reduced patient wait times from their presentation to fracture clinic review [7]. In addition, Khan et al., demonstrated the same efficacy through their high reported range from 46 to 100% of the ability of VFCs in their studies to meet BOAST 72-h guidelines [6].

### VFCs rarely miss injuries or order inappropriate radiographs and have low levels of inappropriate referrals

This paper shows that VFCs rarely miss injuries upon review or order inappropriate radiographs. However, these results are limited to two studies respectively, which reduces their reliability. Nevertheless, these results indicate that VFCs are accurate in providing the correct diagnosis and management to patients when reviewed. Also, the lack of inappropriate radiographs will save valuable time and money for health services across the UK. In addition to this, Little et al. highlights that when VFCs do make diagnostic errors they are effectively resolved through recalling patients back to the fracture clinic [32].

Furthermore, the low levels of inappropriate or rejected referrals indicate appropriate use of the VFC system by healthcare staff which displays that the pathway is well understood. Over time, with appropriate education and further exposure to the system, healthcare staff will become increasingly accustomed to the use of VFCs. Alongside the lack of adverse outcomes in our studies this indicates that VFCs are safe. Murphy et al. supports these conclusions through VFCs in seventeen out of 18 of their studies being deemed safe as well as a five studies reporting a reduction in resources such as radiography use [7]. Khan et al. also states that there were no significant outcomes following VFC consultation in their studies, highlighting their safety [6].

### Reattendance rates following discharge from VFCs are low

The low reattendance rates reported in this paper, following VFC discharge, means that VFCs provide a thorough assessment of patients and are effective in providing individualized care and management plans. However, this cannot always apply to all patients due to interpersonal variability. Atypical patient presentations e.g., increased pain or non-resolving symptoms will always arise, and these patients may reattend. This identifies a downside to VFCs in that they do not involve initial patient contact with a consultant. An initial in person examination on presentation by a consultant could identify these differences and prevent reattendances due to their expertise. However, this defeats the objective of the VFC.

The reduction in non-attends displayed by Nagy et al. with VFC introduction demonstrates the accessibility of this care for patients [5]. Additionally, the reduction in non-attends means that less resources and clinical time are wasted which again contributes to the potential cost savings of the VFC. Khan et al. reviewed a paper which detailed a 75% reduction in non-attends six months after VFC implementation and stated that this reduces cost [6]. However, the fact that 28% of patients in Nagy et al. struggled to attend their FTF appointment highlights accessibility challenges [5]. Patients may struggle to attend FTF appointments due to their injury itself, comorbidities or socio-economic factors. These situations should be identified and addressed within the VFC itself as part of the review and an appropriate action plan should be formulated for their care e.g., providing transport or travelling to the patient.

### VFCs reduce the number of FTF clinic appointments but following VFC review there are high rates of FTF fracture clinic follow ups

The results from this paper display that VFC implementation significantly reduces the number of FTF appointments. This enhances care in FTF appointments through increased time patients spend with their consultants, as well as through a reduction in strain on healthcare services. This enables doctors to have full access to resources and give their undivided attention to patients. Furthermore, reducing FTF appointments will significantly reduce costs for healthcare services. Khan et al. supports these findings through reporting a reduction in resources required across clinics and multi-disciplinary teams which contributed to cost savings [6].

Murphy et al. concurred with these results with 14 of their reviewed studies reporting saved clinical appointments [7]. Furthermore, Khan et al. reported an overall reduction in FTF consultations with VFC implementation [6]. Despite this many patients are in fact recalled through VFCs for in person reviews. Whilst this demonstrates that the correct care and management is being provided for patients through VFC review, it cannot be overlooked that this reveals a major downside to VFCs in that many patients cannot fully be managed virtually. Jain et al. detailed that 48% of their patients who went through VFC review required an in person physical examination to further assess their injury which could not be performed virtually [24]. Although we know that VFCs reduce travel and are more accessible for patients, this shows that many patients in fact still have to present for FTF review.

Interestingly, Sharma et al. recognises the benefits of both FTF clinics and VFCs and demonstrates that perhaps a hybrid fracture clinic combining both is the most effective pathway for care in ATO injuries [8]. However this is a single study and needs further research to confirm the validity of these conclusions [8].

### Patients have low levels of disability, high levels of function and good health outcomes following VFC management

This paper reports that following VFC management, patients have high levels of health according to their EQ-5D-5L scores, which indicates that VFCs are efficacious in providing the correct care and management to patients. However, patients with low EQ-5D-5L scores initially may be more inclined for in person review due to pre-existing co-morbidities whereas patients with higher initial EQ-5D-5L scores may be more suitable for VFC review. These factors question the ability of the EQ-5D-5L score to measure the overall efficacy of VFCs in this situation.

This paper reports low levels of upper limb disability in patients according to their QuickDASH scores, which indicates that VFCs are able to effectively manage upper limb fractures. However, this cohort only captures QuickDASH scores from patients with hand, wrist and clavicle fractures. Therefore, this may not be representative of all upper limb fractures as only a small proportion are assessed.

Furthermore, this paper reports high levels of function after toe and 5th metatarsal base fractures in patients according to their FAAM scores. This demonstrates that VFCs are safe and have the ability to return patients back to their baseline health before these fractures. However, as previously stated, patients with these fractures may be more inclined for DD and can expect good outcomes with minimal orthopaedic input. Therefore, using the FAAM score as an indicator of overall VFC efficacy here may be unreliable.

Unfortunately, neither Khan nor Murphy et al. reported PROMs in their recent systematic reviews [6, 7].

For all 3 PROMs scores, it must be taken into account that there is no comparison between patients scores from before VFC management to afterwards. Therefore, there is no way of quantifying the true effect that VFCs had on the health of these patients.

### Patients managed through the VFC are highly satisfied and prefer VFCs to FTF fracture clinics

This paper shows that patients are highly satisfied with their care following management through VFCs. This means that the care provided by VFCs is high quality and meets the standards set by and the needs of our patients. Murphy et al. reviewed 8 studies that reported VFC patient satisfaction and similar to this paper they found considerable heterogeneity in whether satisfaction related to injury treatment or the VFC process itself [7]. Khan et al. reviewed 4 studies and reported high overall satisfaction rates between 91 and 97% for the VFC process and 85% to 95% for information provided on VFC review, which correlates with our mean overall satisfaction rate of 85.4% [6].

The majority of our included studies that reported comparisons between VFCs’ and FTF facture clinics’ patient satisfaction agreed that currently patients prefer VFCs. This result was surprising as we hypothesized that patients would prefer FTF fracture clinics due to the ability to see a doctor as well as the in person human to human interaction. Given the current climate of the NHS, this trend may be a result of patient preference for the convenience that VFCs offer considering the lack of travel required and prompt follow up appointments. Additionally, this demonstrates the acceptance of the VFC model by patients, as well as the digital evolution of healthcare in the UK. This may be a side effect of the COVID-19 pandemic, changing lay perceptions and normalising virtual care, as all of our comparison studies took place during or after the COVID-19 pandemic.

In addition, by reviewing dissatisfaction rates in our studies, we identified common problems reported by patients that lead to poor experiences with the VFC. These were mainly centred around insufficient communication. Whilst these complaints were few, this displays that VFCs need to improve their ability to communicate care to patients with the aim of all patients understanding their management plan. Potential barriers to effective communication include lack of patient digital access, poor telephone connection or rushed calls. Implementation of communication standards for VFC review telephone calls may help to combat these barriers and minimize suboptimal care.

### Limitations

Several patient population characteristics were not extracted from our studies including the age or sex of patients. Additionally, clinical efficacy, PROMs and patient satisfaction data was not directly compared between different fracture types and fracture complexity. Exploration of these domains may have revealed further trends to be analysed. Fourteen studies did not specify their patient populations which produced some ambiguity among study comparisons. Twenty-three studies were observational and only 2 were closed loop audits. Inclusion of more audits would have been more appropriate for the direct contrast of clinical efficacy and patient satisfaction data between VFCs and FTF fracture clinics.

Adherence of VFCs to BOAST guidelines was only measured using the BOAST 72-h guideline, with the other 12 guidelines not assessed. There was only a small number of studies that assessed adverse outcomes with 2 reporting missed injuries and a lack of data on why patients who reattended did so. Additionally, with only 6 studies reporting PROMs data this is only a small proportion of the 25 included studies. As well as this, there was a narrow range of injuries included in our studies that could be applied to the QuickDASH and FAAM scores.

There was significant heterogeneity between the clinical efficacy data of studies due to differing statistical analysis, study designs, fracture clinic models and types of included injuries as well as varying populations and study timescales.

Furthermore, there was similar heterogeneity in overall patient satisfaction measurements between studies. Different studies measured overall patient satisfaction of the VFC through positive experiences, injury management, clinical outcomes, the VFC process itself or did not specify. Therefore, the satisfaction of patients following VFC management can be attributed to a number of domains: the patient’s clinical outcomes, the VFC process itself or the quality of care they received. Among our studies it was often unclear what satisfaction was relating to. Moreover, there was no standard questionnaire used to quantify patient satisfaction. Each study used a specifically designed questionnaire with different questions and scoring systems which were completed at varying follow-up points.

## Future recommendations

One topic mentioned but not explored in this paper is the ability of VFCs to reduce cost. Murphy et al. and Khan et al. both explore this in their systematic reviews in 2020 [6, 7]. Despite the reduction in cost from VFC implementation having already been researched, a further current cost analysis within the UK itself would be beneficial to further support the transition of the FTF fracture clinic into a standardised VFC model.

Twelve studies that focused only on paediatric patients were excluded during study selection to maintain a balanced patient cohort. These studies could be utilized to address the efficacy of VFCs purely in paediatric patients. Additionally, 28 studies were excluded because they were not set in the UK. These studies could be used to address the suitability and efficacy of VFCs internationally.

Further research could be undertaken to assess the ability of VFCs to adhere to all BOAST guidelines, not just BOAST 72-h guidelines, and into the effect of VFCs on the quality of FTF appointments. The question as to why patients prefer VFCs to FTF fracture clinics needs to be explored to fully understand this transition in patient’s mindsets and their current views on remote care.

A further audit may be beneficial to assess whether there is any discrepancy in patient clinical outcomes. This would focus on where the patient initially presents: the emergency department or MIU and which healthcare staff initially review the patient and their radiographs: nurses, nurse practitioners, junior doctors or consultants.

A study focusing on the ability of vulnerable groups to access VFCs and why they may or may not engage with them would identify targetable areas for improvement and challenge socioeconomic health inequalities.

Building on the 2020 study by Khan et al., a paper taking into account a broader range of different fracture types as well as fracture complexity would prove beneficial [6]. Direct comparison of their efficacy, PROMs and patient satisfaction would provide a useful insight into how fracture complexity affects VFC efficacy as well as patient satisfaction. Also, this would highlight any potential discrepancies in VFC management for particular injuries.

Evaluating, additional PROMs scoring systems would be beneficial to assess the capability of VFCs to manage different types of fractures. Moreover, considering that the universal bias displayed amongst PROMs as a measure of VFC efficacy reduces the reliability of our results, we recommend the need for a uniform validated tool to assess PROMs from VFCs in a way that reduces bias and provides steadfast results.

The main benefits of VFCs include reduced patient wait times, increased patient accessibility, reduced strain on healthcare services and cost savings. With orthopaedic healthcare in the UK adopting a more digital approach could the implementation of artificial intelligence for clerical assistance, triage or radiograph screening be implemented in the future to further reduce patient wait times as well as save resources, clinical time and money.

A review of studies that directly compares clinical efficacy between VFCs and FTF fracture clinics, through the successful management of patients, should be undertaken before a complete transition in the UK.

## Conclusion

This systematic review proves that VFCs are a highly efficacious alternative to the FTF fracture clinic in the UK. With their increasing use across the UK since the COVID-19 pandemic, we found that VFCs can accurately diagnose and manage ATO injuries. In addition, they consistently meet BOAST 72-h guidelines, whereas FTF fracture clinics cannot. Patient satisfaction is very high following management through the VFC, and patients currently prefer VFCs to FTF fracture clinics. The heterogeneity between our included studies and outcome measures means that we cannot conclude that VFCs should become the standard pathway for ATO care across the UK. To make this conclusion further research is needed making direct comparisons between the efficacy of VFCs and FTF fracture clinics. However, this paper does provide comprehensive data to suggest that VFCs could become the leading pathway for ATO care in the future.

## Supplementary Information

Below is the link to the electronic supplementary material.

Supplementary Material 1

## Data Availability

No datasets were generated or analysed during the current study.

## Abbreviations

FTF: Face-to-face
VFC: Virtual fracture clinic
MIU: Minor injuries unit
BOAST: British Orthopaedic Association Standards for Trauma
ATO: Acute traumatic orthopaedic
PRISMA: Preferred Reporting Items for Systematic Reviews and Meta-Analyses
ITS: Interrupted time series
DD: Direct discharge
PROMs: Patient reported outcomes
EQ-5D-5L: EuroQoL Group 5D-5L
QuickDASH: Quick Disabilities of the Arm, Shoulder and Hand
FAAM: Foot and Ankle Ability Measure
NPS: Net promoter score
CES: Customer effort score
CSS: Customer satisfaction score
NOS: Newcastle Ottawa Scale
CE: Clinical efficacy
N.S: Does not specify
PS: Patient satisfaction
