# Supplementary material for: The clinical efficacy and patient satisfaction of the virtual fracture clinic in the UK: a systematic review
Source: J Orthop Surg Res. 2025 Nov 10;20:982. doi: 10.1186/s13018-025-06418-3 (PMC12604321; doi:10.1186/s13018-025-06418-3)
Supplement: Supplementary file 1 — Supplementary Material 1 [file 13018_2025_6418_MOESM1_ESM.docx]

# **Supplementary Material**

|  | **Section** | **Description** | **Paper** | |
| --- | --- | --- | --- | --- |
| 1) | Internal validity |  | Dunkerley et al^(30)^ | Holgate et al^(33)^ |
|  |  |  | Result | |
| 1a) | Bias related to temporal procedure | Is it clear in the study what the cause' and 'effect' is and which one comes first? | Yes | Yes |
| 1b) | Bias related to selection and allocation | Was there a control group? | No | No |
| 1c) | Bias relating to confounding factors | Were participants included in any comparisons similar? | No | No |
| 1d) | Bias related to administration of intervention/exposure | Were participants included in comparisons receiving similar treatment/care other than exposure? | No | No |
| 1e) | Bias related to assessment, detection and measurement of outcome | Were there multiple measurements of the outcomes pre/post intervention? | Yes | Yes |
|  |  | Were the outcomes of participants included in any comparisons measured similarly? | Yes | Yes |
|  |  | Were the outcomes measured in a reliable way? | Yes | Yes |
| 1f) | Bias related to participant retention | Was the follow up complete? If not were differences between groups adequately analyzed? | Yes | Yes |
| 2) | Statistical conclusion validity | Was appropriate statistical analysis used? | Yes | Yes |
|  |  | Overall appraisal | Include | Include |

**Table 9 - JBI Critical Appraisal Checklist for Quasi-experimental studies**

*This table displays the results of bias assessment in our closed loop audits using the JBI Critical Appraisal Checklist for Quasi-experimental studies. The table also includes our overall appraisal of these studies.*
